# Functional Properties of Mouse Chitotriosidase Expressed in the Periplasmic Space of *Escherichia coli*

**DOI:** 10.1371/journal.pone.0164367

**Published:** 2016-10-07

**Authors:** Masahiro Kimura, Satoshi Wakita, Kotarou Ishikawa, Kazutaka Sekine, Satoshi Yoshikawa, Akira Sato, Kazuaki Okawa, Akinori Kashimura, Masayoshi Sakaguchi, Yasusato Sugahara, Daisuke Yamanaka, Naohito Ohno, Peter O Bauer, Fumitaka Oyama

**Affiliations:** 1 Department of Chemistry and Life Science, Kogakuin University, Hachioji, Tokyo, 192–0015, Japan; 2 Laboratory for Immunopharmacology of Microbial Products, Tokyo University of Pharmacy and Life Sciences, Hachioji, Tokyo, 192–0392, Japan; 3 Department of Neuroscience, Mayo Clinic, Jacksonville, FL, 32224, United States of America; USDA-ARS, UNITED STATES

## Abstract

Chitotriosidase (Chit1) is an enzyme associated with various diseases, including Gaucher disease, chronic obstructive pulmonary disease, Alzheimer disease and cystic fibrosis. In this study, we first expressed mouse mature Chit1 fused with V5 and (His)_6_ tags at the C-terminus (Chit1-V5-His) in the cytoplasm of *Escherichia coli* and found that most of the expressed protein was insoluble. In contrast, Chit1 tagged with Protein A at the N-terminus and V5-His at the C-terminus, was expressed in the periplasmic space of *E*. *coli* as a soluble protein and successfully purified. We evaluated the chitinolytic properties of the recombinant enzyme using 4-nitrophenyl *N*,*N’*-diacetyl-β-D-chitobioside [4NP-chitobioside, 4NP-(GlcNAc)_2_] and found that its activity was comparable to CHO cells-expressed Chit1-V5-His. Optimal conditions for the *E*. *coli-*produced Chit1 were pH ~5.0 at 50°C. Chit1 was stable after 1 h incubation at pH 5.0~11.0 on ice and its chitinolytic activity was lost at pH 2.0, although the affinity to chitin remained unchanged. Chit1 efficiently cleaved crystalline and colloidal chitin substrates as well as oligomers of *N*-acetyl-D-glucosamine (GlcNAc) releasing primarily (GlcNAc)_2_ fragments at pH 5.0. On the other hand, (GlcNAc)_3_ was relatively resistant to digestion by Chit1. The degradation of 4NP-(GlcNAc)_2_ and (GlcNAc)_3_ was less evident at pH 7.0~8.0, while (GlcNAc)_2_ production from colloidal chitin and (GlcNAc)_6_ at these pH conditions remained strong at the neutral conditions. Our results indicate that Chit1 degrades chitin substrates under physiological conditions and suggest its important pathophysiological roles *in vivo*.

## Introduction

Chitin is a polymer of β-1, 4-linked *N*-acetyl-D-glucosamine (GlcNAc), and is an integral component of the exoskeletons of crustaceans and insects, the microfilarial sheaths of parasites and the fungal cell walls. Thus, next to cellulose, it is the second most abundant polysaccharide in nature [1, 2].

Chitinases are thought to be essential in chitin digestion by hydrolyzing its β-1, 4 glycoside bonds as a defensive action and/or utilization of chitin as a source of carbon and energy [2–4]. Although mammals do not produce chitin, two active chitinases, chitotriosidase (Chit1) and acidic mammalian chitinase (AMCase), have been identified in mouse and human [3]. Both enzymes show sequence homology to bacterial chitinases and belong to the family 18 of glycosyl hydrolases, which also includes chitinase-like proteins structurally related to chitinases but lacking chitinolytic activity [3, 5–8].

Since Chit1 levels are 1000-fold-elevated in the plasma of patients with Gaucher disease, an autosomal recessive lysosomal storage disorder [9], it was the first mammalian chitinase to be cloned and purified [10, 11]. A 24-bp insertion in exon 10 of the Chit1 gene results in preventing formation of active enzyme [12]. In most ethnic groups, ∼5% of individuals are homozygous for this mutation and therefore lack Chit1 activity. Of note, Chit1 is synthesized by activated human macrophages and neutrophils [9–11, 13]. AMCase was discovered while searching for a compensatory mechanism in Chit1 deficiencies and was named for its acidic isoelectric point [14, 15]. These mammalian chitinases are regarded as part of the host defense mechanism against chitin-containing pathogens and parasites [2, 3].

Chit1 is a secreted protein with a molecular mass of approximately 50 kDa. It consists of an N-terminal catalytic domain (CatD) and a C-terminal chitin-binding domain (CBD) [10]. A shorter Chit1 form of 39-kDa produced in the lysosomes of the macrophages by proteolytic processing of the full length protein retains its chitinase activity [16]. Chit1 and its CatD has a broad pH optimum with the peak at around pH 5 [15, 17].

Chit1 has attracted considerable attention due to its increased expression in individuals with different pathological conditions such as Gaucher disease [9], chronic obstructive pulmonary disease (COPD) [18], Alzheimer’s disease [19], atherothrombosis [20], diabetes mellitus [21] cystic fibrosis [22] as well as in smokers [23]. However, the contribution of Chit1 to the pathogenesis of these diseases and pathophysiological conditions remains to be determined. Recently, we found the highest levels of Chit1 mRNA in mouse stomach, followed by eyes and lungs [24]. We observed this mRNA at low, but readily detectable levels in many other tissues [24, 25]. Further investigation of biomedical roles of Chit1 requires large quantities of purified protein.

Previously, we successfully expressed mouse AMCase as a recombinant protein fused with Protein A, V5 epitope and (His)_6_ tag (V5-His) (Protein A-AMCase-V5-His) in the periplasmic space of *E*. *coli* [26, 27]. In this study, we produced mouse Chit1 as a soluble recombinant fusion of Protein A-Chit1-V5-His, which cleaved crystalline and colloidal chitin substrates as well as GlcNAc oligomers producing primarily (GlcNAc)_2_ fragments at pH 4.0~8.0. Thus, the *E*. *coli*–expressed recombinant mouse Chit1 is sufficient for chitin substrates recognition and degradation in physiological conditions.

## Results

### Expression of mouse Chit1 in the cytoplasm of *E*. *coli* using pET system

**Fig 1:**
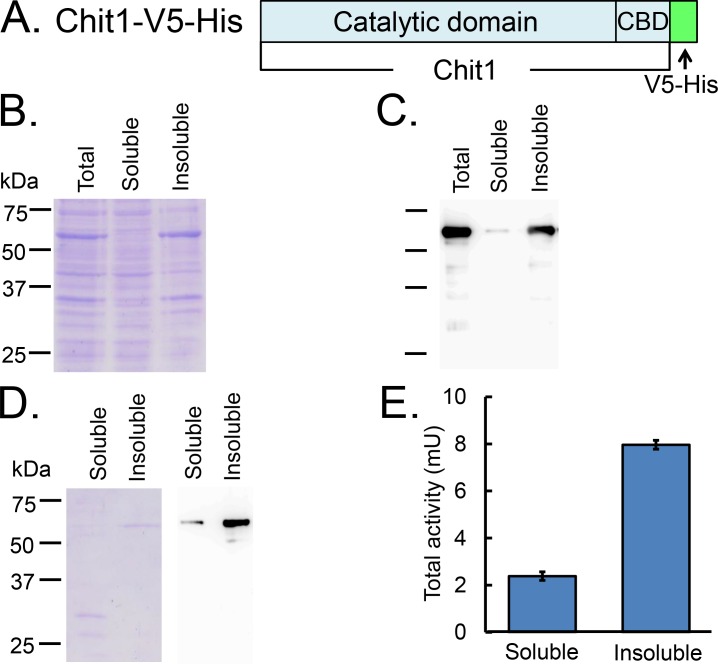
Expression of mouse Chit1 in the cytoplasm of *E*. *coli* using pET system. (A) Schematic representations of the *E*. *coli*-expressed mouse mature Chit1-V5-His. Mouse Chit1 is a secreted protein with a molecular mass of approximately 50 kDa, which contains an N-terminal catalytic domain and a C-terminal chitin-binding domain (CBD). (B) 10% SDS-PAGE of the total *E*. *coli* extract, soluble and insoluble recombinant proteins from *E*. *coli*. The proteins in the gel were visualized by CBB staining. (C) Western blot analysis of the recombinant proteins using anti-V5-antibody. Proteins were run by SDS-PAGE and transferred to a PVDF membrane. (D) Analysis of soluble and insoluble Chit1-V5-His. The recombinant proteins were separated from the soluble fraction by Ni Sepharose. In addition, insoluble-Chit1-V5-His was denatured by 8 M urea and refolded on Ni Sepharose. (E) Comparison of the chitinolytic properties of the purified recombinant proteins from soluble and insoluble fractions in 50 μL reactions using McIlvaine’s buffer (pH 5.0) at 37°C for 30 min as described in the Materials and Methods.

First, we expressed mouse Chit1 in the cytoplasm as mouse mature Chit1-V5-His (Fig 1A) using pET system in *E*. *coli*. For bacterial expression, we subcloned the mature form of Chit1 precursor cDNA into the pET21-d vector as described in Materials and Methods (Fig 1A and S1 Fig). The pET21-d/mature Chit1-V5-His plasmid was tested for its expression in the *E*. *coli* strain BL21 (DE3). We prepared a total *E*. *coli* extract as well as Tris-buffered saline soluble and insoluble fractions and analyzed them by SDS-polyacrylamide gel electrophoresis (PAGE), followed by Coomassie Brilliant Blue (CBB) staining and Western blotting. As shown in Fig 1B and 1C, we detected Chit1-V5-His in both soluble and insoluble fractions, but most of the expressed recombinant proteins were present in the insoluble fraction (Fig 1D and 1E). We purified our fusion protein from the soluble fraction by Ni Sepharose using the His-tag (Fig 1A). In addition, we tried to denature the insoluble-Chit1-V5-His by 8 M urea and refold directly on Ni Sepharose in the insoluble fraction and measured the chitinolytic activity as described in Materials and Methods. Our results indicate that although Chit1-V5-His expressed in the *E*. *coli* cytoplasm has chitinolytic activity, it is mostly present in insoluble form.

### Protein A-Chit1-V5-His fusion protein expressed in the periplasmic space of *E*. *coli* is soluble

**Fig 2:**
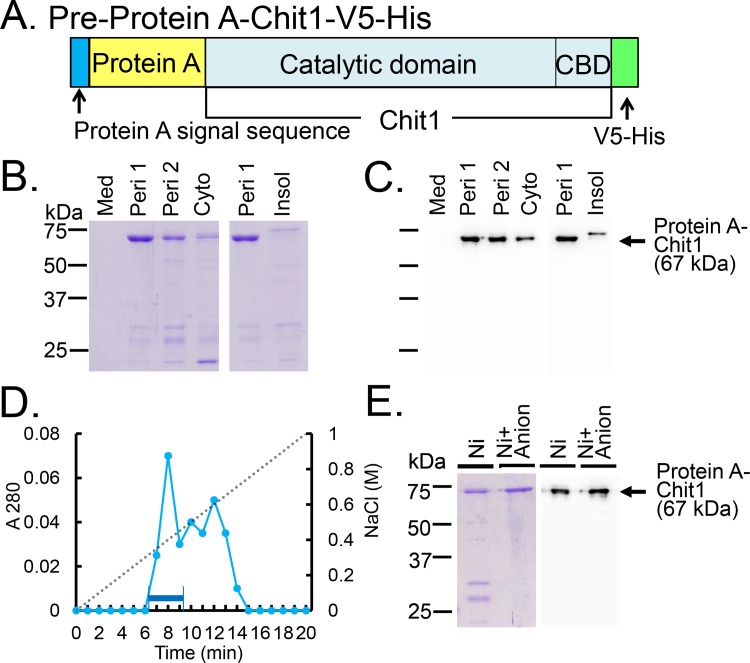
Production of the Chit1 in the periplasmic space of *E*. *coli* as a fusion of Protein A-Chit1-V5-His. (A) Schematic representation of the *E*. *coli*-expressed pre-Protein A-Chit1-V5-His. (B) 10% SDS-PAGE analysis of the recombinant proteins from the culture medium (Med), periplasmic fractions (Peri 1 and Peri 2), cytoplasmic soluble fraction (Cyto) and the insoluble fraction (Insol) from *E*. *coli*. The proteins were visualized by CBB staining. (C) Western blot analysis of the recombinant proteins using anti-V5 antibody. (D) Chromatogram of Protein A-Chit1-V5-His using Hitrap Q HP columns. Bound proteins were eluted with linear gradient of 0 to 1.0 M NaCl. First peak (bold lined) was pooled. (E) SDS-PAGE analysis of the protein fractions purified using the Ni Sepharose, followed by Hitrap Q HP columns. Purified protein were electrophoresed and visualized by staining with CBB. Western blot was performed with anti-V5-HRP antibody.

To enhance the yield of soluble recombinant Chit1, we introduced the Chit1-V5-His cDNA into the pEZZ18 vector [28] containing the *Staphylococcus aureus* Protein A signal sequence. This plasmid was designed to express pre-Protein A-Chit1-V5-His (Fig 2A and S2A Fig) constitutively and to secrete the mature Protein A-Chit1-V5-His (S2B Fig) into the periplasm and subsequently to the culture medium in *E*. *coli* [28].

After transformation of *E*. *coli* with pEZZ18/pre-Protein A-Chit1-V5-His, we first isolated the fusion protein from the culture medium, periplasmic space and cytoplasmic fractions using Ni Sepharose and examined the distribution of the chitinase activity in these compartments. As shown in Table 1, more than 90% of the chitinolytic activity was detected in the periplasmic fractions [periplasmic space 1/osmotic shock (Peri 1) and periplasmic space 2/lysozyme (Peri 2) fractions] of *E*. *coli*. The culture medium and the cytoplasmic soluble fraction contained 0.02% and 7% of the total chitinolytic activity, respectively. We solubilized the insoluble fraction using 8 M urea and performed the refolding and purification of the recombinant protein directly on Ni Sepharose. The chitinolytic activity in this fraction was very low (approximately 1% of the total, Table 1).

**Table 1:** Subcellular distribution of chitinolytic activity of Protein A-Chit1-V5-His in *E*. *coli*.

Fraction	Total activity (mU)	Distribution (%)
Medium	0.1	0.02
Periplasm 1 (Peri 1)	116.2	25.46
Periplasm 2 (Peri 2)	302.1	66.21
Cytoplasm	33.5	7.34
Insoluble	4.5	0.98

Next, we analyzed the expression of Protein A-Chit1-V5-His in four *E*. *coli* fractions separated using Ni Sepharose, by SDS-PAGE and CBB staining. We detected most of the fusion protein (67 kDa; S2B Fig) in the periplasmic (Peri 1 and Peri 2) fractions followed by the cytoplasmic compartment, while there was no corresponding band present in the culture medium (Fig 2B). We confirmed this observation by Western blot using anti-V5 antibody (Fig 2C and S2B Fig). The slower migration of the recombinant protein in the insoluble fraction corresponds to the pre-Protein A-Chit1-His containing signal peptide (Fig 2B and 2C and S2A Fig). These results clearly indicate that most of the chitinase activity was present in the fractions with soluble form of the fusion protein in the periplasmic space of *E*. *coli* (Fig 2B and 2C; Table 1).

Protein A-Chit1-V5-His was separated using Ni Sepharose column and further purified using the HiTrap Q HP column (Fig 2D). The peak fraction (Fig 2D, bold lined) was subjected to SDS-PAGE and analyzed by CBB staining and Western blot. As shown in Fig 2E, we obtained a highly pure Protein A-Chit1-V5-His usable for *in vitro* enzymatic assays.

### Comparison of chitinolytic activity of *E*. *coli*- and CHO-produced Chit1 proteins

**Fig 3:**
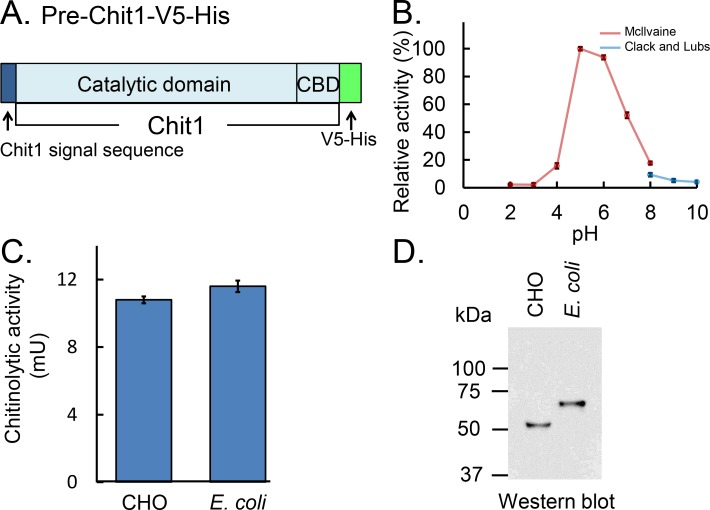
Comparison of the chitinolytic properties of mouse Chit1 prepared from *E*. *coli* and CHO cells. (A) The schematic representations of the CHO-expressed pre-Chit1-V5-His. (B) pH profile of Chit1-V5-His. (C) We first measured the chitinolytic activity of the enzyme preparations from CHO cells and *E*. *coli* in a volume of 50 μL in McIlvaine's buffer (pH 5.0) at 37°C for 30 min. Then we adjusted the enzyme solutions to have same activity. We analyzed the immunoreactivities of these enzymes by Western blot using anti-V5 antibody. (D) The enzyme fractions with the same chitinase activities were visualized by Western blot using anti-V5 antibody. Molecular mass of Protein A-Chit1-V5-His expressed in E. coli was higher than that of Chit1-V5-His. CHO-expressed Chit1 and E. coli-produced Chit1 gave similar signals at approximately 52 kDa and 67 kDa, respectively.

We compared chitin hydrolytic activities of *E*. *coli*-expressed Protein A-Chit1-V5-His and CHO-expressed Chit1-V5-His similarly to our previous study evaluating AMCase [26]. For expression in CHO cells, the entire coding region of Chit1 precursor (pre-Chit1) cDNA was subcloned into the mammalian expression vector pcDNA3.1/V5-His C to produce pre-Chit1-V5-His with a signal sequence at the N-terminal region (Fig 3A and S3A Fig). Expression of this cDNA in CHO cells led to the secretion of mature Chit1-V5-His into culture medium (S3B Fig).

To characterize CHO-expressed Chit1-V5-His, we examined the chitinolytic activity of Chit1-V5-His using 4NP-(GlcNAc)_2_ as a substrate at 37°C and pH ranging from 2.0 to 10.0 for 30 min. As shown in Fig 3B, the recombinant enzyme had highest activity at around pH 5.0~6.0 with a drop at more acidic (pH 4.0) or neutral conditions (pH 7.0~8.0) (Fig 3B).

We next measured the chitinolytic activity of both enzyme preparations using 4NP-(GlcNAc)_2_ and adjusted the enzyme solutions to obtain same activity (Fig 3C). Then, we analyzed the levels of the enzymes in the samples by Western blot using anti-V5 antibody, which recognized the recombinant Chit1 fusion proteins produced in *E*. *coli* and in CHO cells (Figs 2A and 3A). Fig 3C and 3D show that the levels of the both Chit1 forms proteins were similar, suggesting experimental equivalence between the CHO-expressed Chit1-V5-His and *E*. *coli*-produced Protein A-Chit1-V5-His and confirming the suitability of the recombinant protein expressed in *E*. *coli* for functional studies.

### Functional characterization of Protein A-Chit1-V5-His

**Fig 4:**
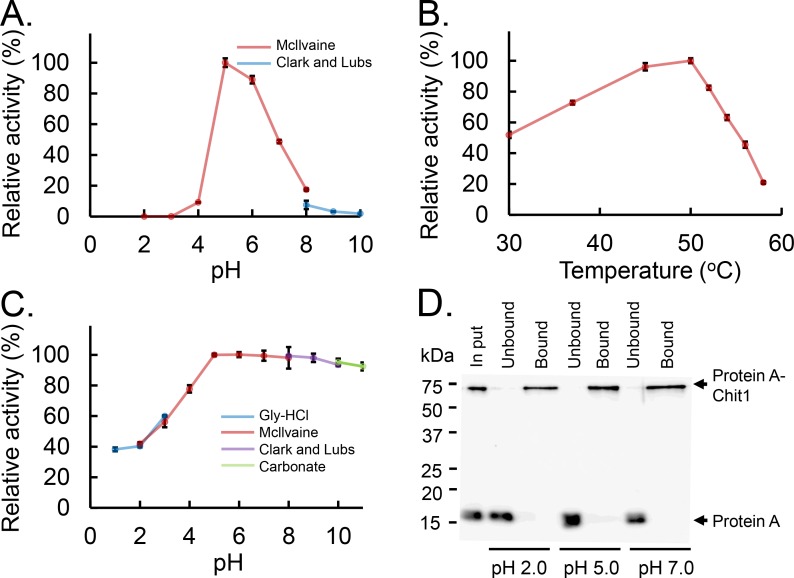
Characterization of the *E*. *coli*-expressed Chit1 activity. (A) pH profile, (B) temperature profile, (C) pH stability profile and (D) affinity of Protein A-Chit1-V5-His to chitin beads. Chitinolytic activity (A~C) was measured as described in the Materials and Methods. The values represent percentage of the maximum activity obtained in each series of experiments. Error bars represent mean ± standard deviation from a single experiment conducted in triplicate. (D) Protein A-Chit1-V5-His and Protein A-V5-His were mixed and loaded onto chitin bead columns. Chitin binding assays were performed at pH 2.0, 5.0 and 7.0 as described in the Materials and Methods. The bound and unbound fractions were analyzed by Western blot using anti-V5 antibody.

To characterize *E*. *coli*-expressed mouse Chit1, we examined the chitinolytic activity of Protein A-Chit1-V5-His using 4NP-(GlcNAc)_2_ as a substrate at 37°C and pH ranging from 2.0 to 10.0 for 30 min. As shown in Fig 4A, the recombinant enzyme had highest activity at pH 5.0 and 6.0 with a drop at more acidic (pH 4.0) or neutral conditions (pH 7.0~8.0) (Fig 4A). Thus, as for the pH preference, *E*. *coli*-expressed Protein A-Chit1-V5-His had properties very similar to CHO-expressed Chit1-V5-His (Fig 3B) as well as COS-1-expressed Chit1-His [15, 17].

The effect of temperature on enzyme activity was determined in McIlvaine buffer at pH 5.0 at 30~58°C using 4NP-(GlcNAc)_2_ for 15 min. The rate of the recombinant Chit1-catalyzed reaction increased with the temperature and reached the maximum level at 50°C, then abruptly declined, indicating denaturation of the protein (Fig 4B).

Next, we determined the pH stability of the recombinant Chit1. The protein was pre-incubated on ice for 1 h at various pH using four different buffers (Fig 4C). The enzymatic activity was then analyzed at 37°C and pH 5.0. As shown in Fig 4C, the recombinant Chit1 retained its full chitinolytic activity over a broad pH range (between 5.0 and 11.0) during the pre-incubation step. Pre-incubation at pH below 5 resulted in reduced chitinolytic activity. Thus, the E. coli-expressed Chit1 is an enzyme able to withstand a broad range of pH (from weak acidic to very basic) conditions.

### Interaction of Protein A-Chit1-V5-His with chitin beads

Full-length Chit1 contains CBD at its C-terminus (Fig 1A). In order to characterize the recognition and interaction of Protein A-Chit1-V5-His with chitin, we carried out a binding assay using chitin-bead columns. We mixed Protein A-Chit1-V5-His and Protein A-V5-His as a control protein [26, 27] at pH 2.0, 5.0 or 7.0 and loaded the samples onto a chitin beads column equilibrated at the pH conditions described in the Materials and Methods. Proteins bound to chitin beads were eluted with 8 M urea [14]. Virtually all Protein A-Chit1-V5-His was detected in the chitin beads-bound fractions, whereas Protein A-V5-His was detected only in the unbound, flow-through fractions at all pH conditions (Fig 4D). These results indicate that *E*. *coli*-expressed Protein A-Chit1-V5-His binds to chitin beads at pH 2.0~7.0, although the chitinolytic activity of the recombinant Chit1 is lost at pH 2.0

### Protein A-Chit1-V5-His cleaves chitin and GlcNAc oligomers

To analyze the chitinolytic activity pattern of Protein A-Chit1-V5-His in detail, we employed chitin (crystalline and colloidal) and (GlcNAc)_3~6_ as substrates. The degradation products were covalently labeled at the reducing end groups with a fluorophore and separated by high-resolution PAGE, as described previously [14, 26, 27, 29].

**Fig 5:**
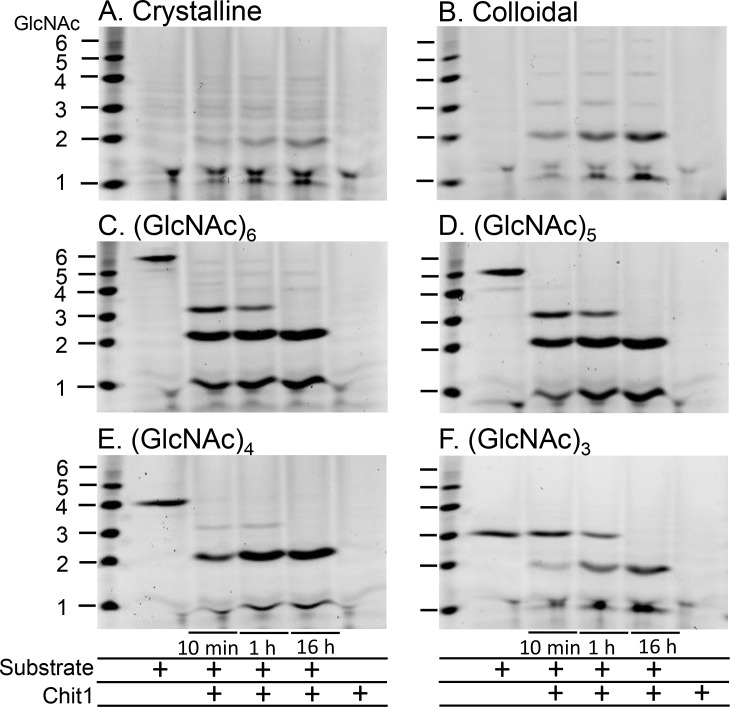
Degradation of colloidal and crystalline chitin and various GlcNAc oligomers by the recombinant Chit1. Crystalline chitin (A), colloidal chitin (B), (GlcNAc)_6_ (C), (GlcNAc)_5_ (D), (GlcNAc)_4_ (E) and (GlcNAc)_3_ (F) were used as substrates in McIlvaine's buffer (pH 5.0). Reactions were conducted for 10 min, 1 h or 16 h at 37°C. Chitin fragments generated by the recombinant proteins were analyzed by fluorophore-assisted carbohydrate electrophoresis. Chitin oligomers are shown in the left margin as standards. Protein A-Chit1-V5-His released primarily (GlcNAc)_2_ fragments from chitin substrates.

First, we incubated crystalline or colloidal chitin at pH 5.0, the optimal pH for Chit1, for 10 min, 1 h or 16 h. We found that Chit1 produced predominantly (GlcNAc)_2_ and low levels of (GlcNAc)_3_ and (GlcNAc)_4_ (Fig 5A and 5B). We next incubated recombinant Chit1 with lower molecular weight GlcNAc oligomers [(GlcNAc)_6,_ (GlcNAc)_5,_ (GlcNAc)_4_ and (GlcNAc)_3_]. As for (GlcNAc)_6_ and (GlcNAc)_5_, we detected two bands corresponding to (GlcNAc)_2_ and (GlcNAc)_3_, latter of which remained stable for 1 h incubation, and then was cleaved to (GlcNAc)_2_ after 16 h incubation (Fig 5C and 5D). Chit1 degraded (GlcNAc)_4_ and produced (GlcNAc)_2_ (Fig 5E).

As for the (GlcNAc)_3_ degradation, not all of the substrate was hydrolyzed by Chit1 within 1 h incubation, in contrast to e.g. (GlcNAc)_4_ (Fig 5F). These results indicate that degradation patterns may be substrate-specific differences between (GlcNAc)_3_ and other oligomers.

### Chit1 degrades chitin substrates in neutral and weak acidic conditions

The results described above indicate that the (GlcNAc)_3_ input substrate as well as the trimer produced from (GlcNAc)_6_ and (GlcNAc)_5_ is relatively resistant to Chit1 digestion. To clarify this finding, we used 4NP-(GlcNAc)_2_, a chromogenic substrate used for evaluating chitinolytic properties of the recombinant enzyme. Since 4NP-(GlcNAc)_2_ is structurally analogous to the (GlcNAc)_3_, their cleavage dynamics is expected to be similar.

**Fig 6:**
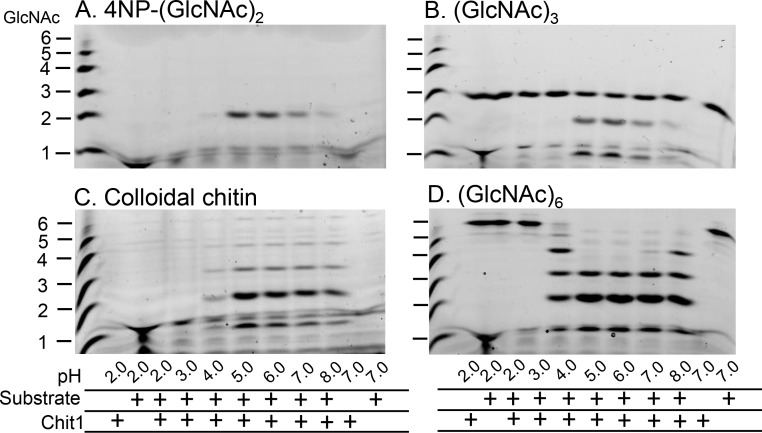
The pH-dependent chitin degradation by the recombinant Chit1. Chit1 chitinase activity was investigated by incubating the enzyme with 4NP-(GlcNAc)_2_ (A), (GlcNAc)_3_, (B), colloidal chitin (C) or (GlcNAc)_6_ (D) as substrates at pH 2.0~8.0. Reactions were conducted for 30 min at 37°C, followed by labeling with a fluorophore and separated by PAGE as described in Materials and Methods. Chitin oligomers are shown in the left margin as standards.

We incubated recombinant Chit1 with 4NP-(GlcNAc)_2_, (GlcNAc)_3_, colloidal chitin or (GlcNAc)_6_ at pH ranging from 2.0 to 8.0 at 37°C for 30 min. Cleavage of 4NP-(GlcNAc)_2_ resulted in strong (GlcNAc)_2_ signals at pH 5.0 and 6.0, which were reduced at higher pH of 7.0 and 8.0 (Fig 6A). These results were consistent with the colorimetric analysis using 4NP-(GlcNAc)_2_ at 405 nm (Fig 4A). Similar results were obtained with (GlcNAc)_3_ with evident resistance of the substrate (Fig 6B) as well as with colloidal chitin and (GlcNAc)_6_ (Fig 6C and 6D). However, the larger substrates were efficiently degraded to (GlcNAc)_2_ at broader pH range (from 4.0 to 8.0) as compared to for 4NP-(GlcNAc)_2_ and (GlcNAc)_3_ (Fig 6C and 6D).

Our results indicate that Chit1 can degrade chitin substrates at pH 4.0~8.0 and produce (GlcNAc)_2_, with preference toward higher molecular weight substrates. This is true especially at pH 7.0 and 8.0, where the level of the (GlcNAc)_2_ has been underestimated when we evaluated the chitinolytic activity using 4NP-(GlcNAc)_2_ substrate. Importantly, elevated levels of degradation at pH 7.0 and 8.0 indicate that Chit1 functions efficiently under physiological pH conditions.

## Discussion

Chit1 is thought to play important roles in pathogenesis of different diseases, such as Gaucher disease, COPD, Alzheimer’s disease and cystic fibrosis [9, 18, 19, 21–23, 30]. However, detailed knowledge on its pathophysiological functions in mice and humans is still unclear. Large quantities of functional Chit1 are required for studying these functions and biochemical characterization. Currently, structural and biochemical characterization of Chit1 relies on mammalian and yeast cell expression systems [15, 17, 31, 32]. Maintenance of *E*. *coli* is simple, quick and inexpensive and can be easily scaled up for recombinant proteins production. Here, we described an *E*. *coli*-expression system allowing periplasmic production of soluble mouse Chit1 with chitinolytic activity comparable to Chit1 expressed in mammalian cells. In addition, we showed that recombinant Chit1 degrades chitin substrates under neutral as well as weak acidic conditions.

We expressed mouse Chit1 in the cytoplasm of *E*. *coli* as mature Chit1-V5-His using pET system. Most of the expressed protein was insoluble (Fig 1B, 1C and 1D). It is well known that a portion of His-tagged proteins can be pelleted after centrifugation of the disrupted cells [33]. Indeed, our previous research has shown that glucoamylase of *Caulobacter crescentus* CB15 formed inclusion bodies in the pET system in *E*. *coli* [34]. Therefore, we used the pEZZ18 vector [28], a Protein A gene fusion vector system based on two synthetic IgG-binding domains (ZZ) of *Staphylococcus aureus* Protein A which has been used for extracellular expression of secretory proteins and for short proteins [35–37]. Using pEZZ18, we previously succeeded to express functional glucoamylase and mouse AMCase in *E*. *coli* [26, 34]. In this study we successfully expressed the functional Protein A-Chit1-V5-His recombinant protein in the periplasmic space using the same *E*. *coli* strain.

Due to the presence of Protein A signal sequence, the fusion proteins are secreted into culture medium. Most of the expressed Protein A-Chit1-V5-His was located in the periplasmic fraction of *E*. *coli* (Fig 2 and Table 1). As shown in Figs 3B and 4A, CHO-expressed Chit1-V5-His and *E*. *coli*-expressed Protein A-Chit1-V5-His showed same pH optimal profiles, which were consistent with those of the protein expressed in COS-1 cells [15, 17]. Thus, one can assume that Chit1 expressed in the periplasmic space tends to form an active tertiary structure identical to that of the naturally synthesized mouse Chit1. Our results indicate that the primary structure of Chit1 is sufficient to form a proper tertiary structure regardless of the Protein A presence. This could be a preserved event due to a conserved sequence among ancient chitinase family [7] and/or periplasmic expression.

We compared the recovery of total chitinolytic activity of *E*. *coli-* and CHO-produced Chit1 estimated for 1 L culture (S1 Table). Yield of our *E*. *coli*-produced Chit1 was not so high when compared to that from CHO cells. We expressed mouse Chit1 as a soluble protein in the culture medium using the constitutive Protein A promoter, whose activity is not very high [28]. If more protein for further biochemical analysis is needed, sufficient amounts of the recombinant Chit1 could be obtained easily by increasing the culture volume.

Protein A-Chit1-V5-His was not secreted into the culture medium (Table 1), whereas small part of Protein A-AMCase-V5-His is present in the medium [26]. This results indicate that AMCase and Chit1 show distinct distribution patterns due to differences in molecular structures although both enzymes belong to family 18 of glycosyl hydrolases [3, 7].

Since recombinant AMCase showed profound acid stability at pH 1 to 3, we could use IgG Sepharose as an affinity chromatography resin for purification of the recombinant AMCase [26, 27]. The bound protein should be eluted with 0.1 M Gly-HCl (pH 2.5). This method can only be used if the fusion product is stable under these conditions. Here, we set different strategy for purification of recombinant Chit1, since this protein is unstable at pH lower than 4. As shown in S4 Fig, AMCase was stable at pH 2.5 for 10 min, whereas 40% of the Chit1 activity was decreased under this condition. Thus, we purified Protein A-Chit1-V5-His using Ni Sepharose. The slight protein contamination was then removed by HiTrap Q column (Fig 2E). Our present purification protocol using Ni Sepharose, followed by HiTrap Q columns is effective for purification of acid-labile proteins including Chit1.

We recently reported that Chit1 mRNA is synthesized at high levels in mouse stomach [24]. Recombinant Chit1 retained its full chitinolytic activity at pH 5.0~11.0 at 0°C for 1 h but it was gradually reduced in response to decreased pH (Fig 4C). In contrast, the recombinant protein did bind to chitin beads at pH 2.0 as well as pH 5.0 and 7.0 (Fig 4D). These results suggest that Chit1 can serve as a lectin binding with chitin substrates in the gastrointestinal environments in mouse.

Chit1 cleaved crystalline and colloidal chitin substrates as well as GlcNAc oligomers and released primarily (GlcNAc)_2_ fragments at pH 5.0. The enzyme digested (GlcNAc)_6_ and (GlcNAc)_5_ producing (GlcNAc)_2_ and (GlcNAc)_3_, latter being stable for 1 h incubation at 37°C, and cleaved to (GlcNAc)_2_ and GlcNAc monomer after 16 h incubation. In contrast, Chit1 efficiently degraded (GlcNAc)_4_ to (GlcNAc)_2_. Our results indicate substrate preference of Chit1 toward larger oligomers and higher molecular weight chitin molecules.

The (GlcNAc)_3_ produced from (GlcNAc)_6_ and (GlcNAc)_5_ was also relatively resistant to the enzyme digestion. This could be due to the inherent degradation property of (GlcNAc)_3_ by Chit1 digestion. 4NP-(GlcNAc)_2_, usually used as a synthetic chromogenic substrate for the detection of chitinolytic activity, showed similar dynamics of digestion by Chit1 as (GlcNAc)_3_ (Fig 6A and 6B) where we detected strong (GlcNAc)_2_ signal at pH 5.0~6.0, but weak bands at pH 7.0~8.0. In contrast, Chit1 degraded colloidal chitin and (GlcNAc)_6_ and produced large amount of (GlcNAc)_2_ at pH 4.0, 7.0 and 8.0 (Fig 6C and 6D). Our present results indicate that Chit1 can degrade chitin substrates at pH 4.0~8.0 and produce (GlcNAc)_2_, especially at pH 7.0 and 8.0.

Recombinant mouse Chit1 is stable and most active at pH 5.0, which reflects the lysosomal acidity (Fig 4A and 4C). In lysosomes of macrophages, the full length 50-kDa Chit1 has been shown to be processed into the 39-kDa form [16]. We show in this paper that Chit1 degrades chitin substrates in neutral as well as weak acidic conditions. Thus, this enzyme can be active and play important roles at physiological conditions in vivo. Chit1 is highly expressed in eyes, suggesting roles in anti-chitin-containing microbial spectrum [38]. Chitinase activity is increased in atherosclerotic patient sera and is present in atherosclerotic plaques. The activity of this enzyme has been shown to have protective role against atherosclerosis indicating that enhancing local chitinase activity may provide a novel treatment for atherosclerosis [39]. Further studies will be needed for detailed analysis of the enzymatic properties of our recombinant Chit1 toward its substrates.

## Materials and Methods

### Mammalian cell expression construct

We used mouse stomach total RNA from the Mouse Total RNA Master Panel (Clontech Laboratories) cDNA production, as previously described [24]. To express mouse Chit1 precursor-V5-His fusion protein (pre-Chit1-V5-His, Fig 3A), Chit1 cDNA (GenBank accession number AY458654.1 nucleotides 229~1620) was amplified from the mouse stomach cDNA by PCR using KOD Plus DNA polymerase (Toyobo) and primers (Sigma-Aldrich Life Science Japan) anchored with the restriction sites for EcoRI and XhoI. The forward primer (5’-**CATG**GAATTCGGAACAAGTTGTAGAGCTCTCGGCT-3) contains 6 bases long EcoRI recognition sequence (underlined) and 25 bases long Chit1 sequence corresponded to nucleotides of the Chit1 cDNA. The reverse primer (5’-**GTGAC**CTCGAGCGCTCCAGGTACAACATTTGCAAG-3’) contains the XhoI recognition sequence (underlined) and is complementary to nucleotides of the Chit1 cDNA and both primers contain the 4~5 bases long extra nucleotides (boldfaced) for efficient cleavage of the PCR products’ termini restriction enzymes. The PCR product was purified using Wizard SV Gel and PCR Clean-Up System (Promega) and digested with EcoRI and XhoI. The cleaved DNA fragment was purified from 1.5% agarose gel and subcloned into a similarly digested pcDNA3.1/V5-His C vector (Invitrogen) (http://tools.invitrogen.com/content/sfs/vectors/pcdna3_1v5hisc_seq.txt). We designed the reverse primer, which is in frame with the N-terminal region of V5-His of pcDNA3.1/V5-His C vector. The entire nucleotide sequence of the resulting plasmid DNA (the pcDNA3.1/pre-Chit1-V5-His) was confirmed by sequencing (Eurofins Genomics).

### *E*. *coli* expression constructs

The mature Chit1-V5-His cDNA region without its signal sequence was amplified from the pcDNA3.1/pre-Chit1-V5-His by PCR using KOD Plus DNA polymerase and primers anchored with EcoRI and SalI restriction sites. The forward primer (5’- **CGCGGAT**GAATTCGAGCAAAACTGGTCTGCTACCTCACCAA-3’) is in frame with the carboxyl terminal region of T7 Tag, and nucleotides of the Chit1 cDNA. The reverse primer (5’-**AGGG**GTCGACTAGAAGGCACAGTCGAGGCTGATCA-3’) is complementary to nucleotides of pcDNA3.1/V5-His C vector. Protein expression vector pET-21d (Novagen) was linearized with EcoRI and SalI, and ligated to the mature Chit1-V5-His cDNA using T4 DNA ligase (Toyobo).

For the production of Protein A-fusion protein, we used the forward primer (5’-**CATG**GAATTCGGCAAAACTGGTCTGCTACCTCACC-3’) containing EcoRI recognition site, which is in frame with the C-terminal region of Protein A, and the Chit1 cDNA. The reverse primer containing SalI recognition site was complementary to nucleotides of pcDNA3.1/V5-His C vector as described above. Protein A fusion vector pEZZ18 (GE Healthcare) was cleaved with EcoRI and SalI, and ligated with mature Chit1-V5-His cDNA using T4 DNA ligase to *E*. *coli* BL21 (DE3) strain (Takara Bio) was used for expression of these plasmids.

### Preparation of soluble and insoluble fractions of *E*. *coli* expressing Chit1-V5-His

Transformed *E*. *coli* were grown in 1 L LB medium containing 100 μg/mL ampicillin at 37°C. After induction with isopropyl β-D-thiogalactopyranoside (IPTG) at OD660 = 0.6, the bacteria were further cultured for 3 h in LB medium. Bacteria were harvested by centrifugation at 5,000 x *g* for 20 min at 4°C. Cells were suspended in 20 mL of 20 mM Tris-HCl (pH 7.6), 0.5 M NaCl containing a protease inhibitor (Complete, Roche), sonicated on ice for 10 min (total extract fraction) and centrifuged at 15,000 x *g* for 20 min at 4°C and the supernatant was pooled (cytoplasmic soluble fraction). The soluble fraction was applied to a Ni Sepharose (GE Healthcare) column equilibrated with 20 mM Tris-HCl (pH 7.6), 0.5 M NaCl. The column was washed with 10-column volumes of 0.05 M imidazole, 0.5 M NaCl in 20 mM Tris-HCl (pH 7.6) and proteins were eluted with 0.5 M imidazole, 0.5 M NaCl in 20 mM Tris-HCl (pH 7.6). The active fractions were desalted with PD10 (GE Healthcare) equilibrated with the TS buffer [20 mM Tris-HCl (pH 7.6), 150 mM NaCl].

The insoluble fraction was solubilized in 8 M urea in 20 mM Tris-HCl (pH 7.6), 0.5 M NaCl solution containing protease inhibitor for 30 min at 4°C. The samples were then centrifuged at 15,000 x *g* for 20 min at 4°C and the supernatants were pooled (solubilized “insoluble fraction”). We solubilized the insoluble fraction using 8 M urea. Refolding and purification of the denatured recombinant protein were then performed on a Ni Sepharose column. The solubilized fraction was applied to a Ni Sepharose column and the His-tagged protein was captured. The column was washed using 10-column volumes of 8 M urea in 20 mM Tris-HCl (pH 7.6), 0.5 M NaCl. Then, the resin was washed with 10-column volumes of 0.05 M imidazole, 0.5 M NaCl in 20 mM Tris-HCl (pH 7.6). Bound proteins were eluted with 0.5 M imidazole, 0.5 M NaCl in 20 mM Tris-HCl (pH 7.6) and desalted as described above.

### Preparation of Protein A-Chit1-V5-His from medium, periplasmic space, soluble and insoluble fractions of *E*. *coli*

Preparation of the recombinant protein from the *E*. *coli* was performed essentially as described previously [26].

### Further purification of Protein A-Chit1-V5-His by HiTrap Q HP column

Two fractions of periplasmic space 1 and 2 were combined and applied to a Ni Sepharose column equilibrated with 20 mM Tris-HCl (pH 7.6), 0.5 M NaCl. The column was washed with 10-column volumes of 0.05 M imidazole, 0.5 M NaCl in 20 mM Tris-HCl (pH 7.6) and proteins were eluted with 0.5 M imidazole, 0.5 M NaCl in 20 mM Tris-HCl (pH 7.6). The peak fraction was diluted with 10 volumes of 20 mM Tris-HCl (pH 7.6) and applied on the HiTrap Q HP (GE Healthcare) equilibrated with same buffer. The column was washed with 10 volumes of 20 mM Tris-HCl (pH 7.6) and eluted with linear gradient of 0 to 1.0 M NaCl. The active fractions were desalted as described above.

### Protein determination, SDS-PAGE and Western blotting

Protein concentrations were determined by the Protein Assay (Bio-Rad) based on the method of Bradford [40] with bovine serum albumin as a standard. The obtained protein fractions were analyzed using standard SDS-PAGE. The proteins in the gel were visualized by Coomassie Blue R-250 staining (Sigma-Aldrich). Separated proteins were transferred to a polyvinylidene fluoride (PVDF) membrane (Immobilon-P, Millipore), which was incubated with an anti-V5-HRP monoclonal antibody (Invitrogen) and Peroxidase AffiniPure F (ab')_2_ Fragment Donkey Anti-Mouse IgG (H+L) (Jackson ImmunoResearch). We used All Blue molecular weight marker (Bio-Rad).

### Enzymatic and chitin binding assays

Chitinolytic activity of Protein A-Chit1-V5-His was determined using the synthetic chromogenic substrate, 4-nitrophenyl *N*,*N′*-diacetyl-β-D-chitobioside [4NP-(GlcNAc)_2_] (Sigma-Aldrich) as described previously [26, 27]. Chitin binding assay were performed as described previously [27] except for using McIlvaine buffer at pH 2.0, 5.0 and 7.0.

### Transient transfection and purification of Chit1-V5-His from culture medium

CHO-K1 cells (CCL61, ATCC) were maintained in Minimum Essential Medium (Invitrogen) supplemented with 10% fetal bovine serum (Biowest). CHO cells were transfected with the Chit1-V5-His expression plasmid using Lipofectamine plus (Invitrogen) according to the manufacturer’s instruction. After 48 h, cell culture media was collected. Secreted Chit1-V5-His was bound to Ni Sepharose equilibrated with 20 mM Tris-HCl (pH 7.6), 0.5 M NaCl. Chit1-V5-His was eluted with 0.5 M imidazole, 0.5 M NaCl and desalted by PD MidiTrap G-25 (GE healthcare) equilibrated with the TS buffer as described above.

### Degradation of chitin substrates by Protein A-Chit1-V5-His

We used shrimp shell chitin (Sigma-Aldrich) as a crystalline chitin. Colloidal chitin was prepared from the chitin, as described previously, and used as a substrate to determine the chitinase activity [14]. All enzymatic reactions using crystalline chitin (1 mg/reaction), colloidal chitin (at a final concentration of 1 mg/mL) and (GlcNAc)_3~6_ (at a final concentration of 200 μM) as substrates were carried out in a volume of 50 μL containing *E*. *coli*-expressed Chit1 in McIlvaine buffer (pH 5.0) essentially as described above except that we used the chitin substrates instead of chromogenic substrate. The reactions were incubated for 10 min, 1 h or 16 h at 37°C.

The pH-dependence of the recombinant Chit was investigated by incubating the enzyme with 4NP-(GlcNAc)_2_, (GlcNAc)_3_ and (GlcNAc)_6_ (at a final concentration of 200 μM) as well as colloidal chitin (at a final concentration of 1 mg/mL) at pH 2.0~8.0. Reactions were conducted for 30 min at 37°C.

The chitin fragments generated from these reactions were labeled covalently at their reducing end groups with the fluorophore 8-aminonaphthalene-1,3,6-trisulphonic acid (ANTS, Invitrogen), and the resulting fluorescent derivatives were separated by high-resolution PAGE, as described by Jackson [29] except for the addition of 5 μL of glacial acetic acid before ANTS labeling. GlcNAc oligomers (Seikagaku Corporation) were used as a standard.

## Supporting Information

S1 Fig Deduced amino acid sequences and their theoretical molecular masses of Chit1-V5-His. The amino acid sequences are color coded, consistent with Fig 1A. Rich Blue, T7 Tag; Blue, mouse mature Chit1; Green, V5-His sequence.
(DOC)

S2 Fig Deduced amino acid sequences and their molecular masses of Protein A-Chit1-V5-His. The amino acid sequences are color coded, consistent with Fig 2A. Rich blue, signal sequence of Protein A; Yellow, truncated form of Protein A; Blue, mouse mature Chit1; Green, V5-His sequence.
(DOC)

S3 Fig Deduced amino acid sequences and their theoretical molecular masses of pre-Chit1-V5-His and mature-Chit1-V5-His. The amino acid sequences are color coded, consistent with Fig 3A. Rich Blue, signal sequence of mouse Chit1; Blue, mouse mature Chit1; Green, V5-His sequence.
(DOC)

S4 Fig Effect of pH 2.5 on the chitinolytic activity of AMCase or Chit1. Protein A-AMCase-V5-His (A) or Protein A-Chit1-V5-His (B) was incubated with 0.1 M Gly-HCl at pH 2.5 for 10 min at room temperature, followed by neutralization with 1 M Tris-HCl (pH 7.6). Then chitinolytic activity was measured at their optimal conditions.
(TIF)

S1 Table Comparison of the total chitinolytic activity of *E-coli-* and CHO-expressed recombinant Chit1. We expressed Protein A-Chit1-V5-His in *E*. *coli* or Chit1-V5-His in CHO cells and measured their chitinolytic activity as described in Materials and Methods.
(DOC)
