# Supplementary material for: Functional Properties of Mouse Chitotriosidase Expressed in the Periplasmic Space of Escherichia coli
Source: PLoS One. 2016 Oct 7;11(10):e0164367. doi: 10.1371/journal.pone.0164367 (PMC5055312; doi:10.1371/journal.pone.0164367)
Supplement: S1 Fig — The amino acid sequences are color coded, consistent with Fig 1A. Rich Blue, T7 Tag; Blue, mouse mature Chit1; Green, V5-His sequence. (DOC) [file pone.0164367.s001.doc]

**Chit1-V5-His**

**489 amino acids 53,961 dalton**

**MASMTGGQQMGRIRIRAKLVCYLTNWSQYRTEAVRFFPRDVDPNLCTHVIFAFAGMDNHQLSTVEHNDELLYQELNSLKTKNPKLKTLLAVGGWTFGTQKFTDMVATASNRQTFVKSALSFLRTQGFDGLDLDWEFPGGRGSPTVDKERFTALIQDLAKAFQEEAQSSGKERLLLTAAVPSDRGLVDAGYEVDKIAQSLDFINLMAYDFHSSLEKTTGHNSPLYKRQGESGAAAEQNVDAAVTLWLQKGTPASKLILGMPTYGRSFTLASSSDNGVGAPATGPGAPGPYTKDKGVLAYYEACSWKERHRIEDQKVPYAFQDNQWVSFDDVESFKAKAAYLKQKGLGGAMVWVLDLDDFKGSFCNQGPYPLIRTLRQELNLPSETPRSPEQIIPEPRPSSMPEQGPSPGLDNFCQGKADGVYPNPGDESTYYNCGGGRLFQQSCPPGLVFRASCKCCTWSARGHPFEGKPIPNPLLGLDSTRTGHHHHHH**
