# Supplementary material for: Functional Properties of Mouse Chitotriosidase Expressed in the Periplasmic Space of Escherichia coli
Source: PLoS One. 2016 Oct 7;11(10):e0164367. doi: 10.1371/journal.pone.0164367 (PMC5055312; doi:10.1371/journal.pone.0164367)
Supplement: S2 Fig — The amino acid sequences are color coded, consistent with Fig 2A. Rich blue, signal sequence of Protein A; Yellow, truncated form of Protein A; Blue, mouse mature Chit1; Green, V5-His sequence. (DOC) [file pone.0164367.s002.doc]

1. **Pre-Protein A-Chit1-V5-His**

**636 amino acids 70194 dalton**

**MKKKNIYSIRKLGVGIASVTLGTLLISGGVTPAANAAQHDEAVDNKFNKEQQNAFYEILHLPNLNEEQRNAFIQSLKDDPSQSANLLAEAKKLNDAQAPKVDNKFNKEQQNAFYEILHLPNLNEEQRNAFIQSLKDDPSQSANLLAEAKKLNDAQAPKVDANSAKLVCYLTNWSQYRTEAVRFFPRDVDPNLCTHVIFAFAGMDNHQLSTVEHNDELLYQELNSLKTKNPKLKTLLAVGGWTFGTQKFTDMVATASNRQTFVKSALSFLRTQGFDGLDLDWEFPGGRGSPTVDKERFTALIQDLAKAFQEEAQSSGKERLLLTAAVPSDRGLVDAGYEVDKIAQSLDFINLMAYDFHSSLEKTTGHNSPLYKRQGESGAAAEQNVDAAVTLWLQKGTPASKLILGMPTYGRSFTLASSSDNGVGAPATGPGAPGPYTKDKGVLAYYEACSWKERHRIEDQKVPYAFQDNQWVSFDDVESFKAKAAYLKQKGLGGAMVWVLDLDDFKGSFCNQGPYPLIRTLRQELNLPSETPRSPEQIIPEPRPSSMPEQGPSPGLDNFCQGKADGVYPNPGDESTYYNCGGGRLFQQSCPPGLVFRASCKCCTWSARGHPFEGKPIPNPLLGLDSTRTGHHHHHH**

1. **Mature Protein A-Chit1-V5-His**

**600 amino acids 66,568 dalton**

**AQHDEAVDNKFNKEQQNAFYEILHLPNLNEEQRNAFIQSLKDDPSQSANLLAEAKKLNDAQAPKVDNKFNKEQQNAFYEILHLPNLNEEQRNAFIQSLKDDPSQSANLLAEAKKLNDAQAPKVDANSAKLVCYLTNWSQYRTEAVRFFPRDVDPNLCTHVIFAFAGMDNHQLSTVEHNDELLYQELNSLKTKNPKLKTLLAVGGWTFGTQKFTDMVATASNRQTFVKSALSFLRTQGFDGLDLDWEFPGGRGSPTVDKERFTALIQDLAKAFQEEAQSSGKERLLLTAAVPSDRGLVDAGYEVDKIAQSLDFINLMAYDFHSSLEKTTGHNSPLYKRQGESGAAAEQNVDAAVTLWLQKGTPASKLILGMPTYGRSFTLASSSDNGVGAPATGPGAPGPYTKDKGVLAYYEACSWKERHRIEDQKVPYAFQDNQWVSFDDVESFKAKAAYLKQKGLGGAMVWVLDLDDFKGSFCNQGPYPLIRTLRQELNLPSETPRSPEQIIPEPRPSSMPEQGPSPGLDNFCQGKADGVYPNPGDESTYYNCGGGRLFQQSCPPGLVFRASCKCCTWSARGHPFEGKPIPNPLLGLDSTRTGHHHHHH**
