# Supplementary material for: Functional Properties of Mouse Chitotriosidase Expressed in the Periplasmic Space of Escherichia coli
Source: PLoS One. 2016 Oct 7;11(10):e0164367. doi: 10.1371/journal.pone.0164367 (PMC5055312; doi:10.1371/journal.pone.0164367)
Supplement: S3 Fig — The amino acid sequences are color coded, consistent with Fig 3A. Rich Blue, signal sequence of mouse Chit1; Blue, mouse mature Chit1; Green, V5-His sequence. (DOC) [file pone.0164367.s003.doc]

1. **Pre-Chit1-V5-His**

**493 amino acids 54,447 dalton**

**MVQSLAWAGVMTLLMVQWGSAAKLVCYLTNWSQYRTEAVRFFPRDVDPNLCTHVIFAFAGMDNHQLSTVEHNDELLYQELNSLKTKNPKLKTLLAVGGWTFGTQKFTDMVATASNRQTFVKSALSFLRTQGFDGLDLDWEFPGGRGSPTVDKERFTALIQDLAKAFQEEAQSSGKERLLLTAAVPSDRGLVDAGYEVDKIAQSLDFINLMAYDFHSSLEKTTGHNSPLYKRQGESGAAAEQNVDAAVTLWLQKGTPASKLILGMPTYGRSFTLASSSDNGVGAPATGPGAPGPYTKDKGVLAYYEACSWKERHRIEDQKVPYAFQDNQWVSFDDVESFKAKAAYLKQKGLGGAMVWVLDLDDFKGSFCNQGPYPLIRTLRQELNLPSETPRSPEQIIPEPRPSSMPEQGPSPGLDNFCQGKADGVYPNPGDESTYYNCGGGRLFQQSCPPGLVFRASCKCCTWSARGHPFEGKPIPNPLLGLDSTRTGHHHHHH**

1. **Mature Chit1-V5-His**

**473 amino acids 52,186 dalton**

**AKLVCYLTNWSQYRTEAVRFFPRDVDPNLCTHVIFAFAGMDNHQLSTVEHNDELLYQELNSLKTKNPKLKTLLAVGGWTFGTQKFTDMVATASNRQTFVKSALSFLRTQGFDGLDLDWEFPGGRGSPTVDKERFTALIQDLAKAFQEEAQSSGKERLLLTAAVPSDRGLVDAGYEVDKIAQSLDFINLMAYDFHSSLEKTTGHNSPLYKRQGESGAAAEQNVDAAVTLWLQKGTPASKLILGMPTYGRSFTLASSSDNGVGAPATGPGAPGPYTKDKGVLAYYEACSWKERHRIEDQKVPYAFQDNQWVSFDDVESFKAKAAYLKQKGLGGAMVWVLDLDDFKGSFCNQGPYPLIRTLRQELNLPSETPRSPEQIIPEPRPSSMPEQGPSPGLDNFCQGKADGVYPNPGDESTYYNCGGGRLFQQSCPPGLVFRASCKCCTWSARGHPFEGKPIPNPLLGLDSTRTGHHHHHH**
