# Supplementary figures and images for: Functional Properties of Mouse Chitotriosidase Expressed in the Periplasmic Space of Escherichia coli
Source: PLoS One. 2016 Oct 7;11(10):e0164367. doi: 10.1371/journal.pone.0164367 (PMC5055312; doi:10.1371/journal.pone.0164367)

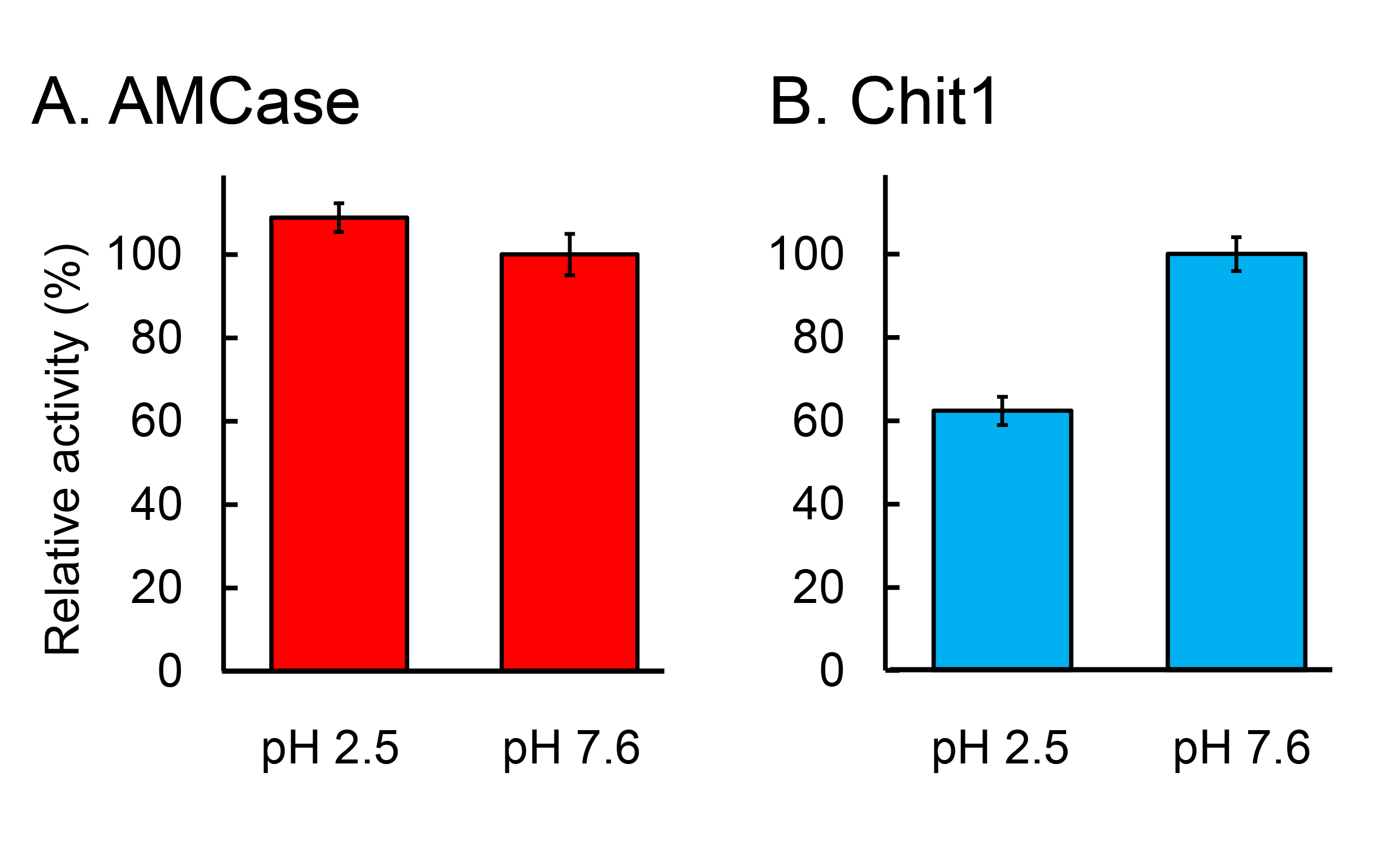

Supplement: S4 Fig — Protein A-AMCase-V5-His (A) or Protein A-Chit1-V5-His (B) was incubated with 0.1 M Gly-HCl at pH 2.5 for 10 min at room temperature, followed by neutralization with 1 M Tris-HCl (pH 7.6). Then chitinolytic activity was measured at their optimal conditions. (TIF) [file pone.0164367.s004.tif]
