# Supplementary material for: Functional Properties of Mouse Chitotriosidase Expressed in the Periplasmic Space of Escherichia coli
Source: PLoS One. 2016 Oct 7;11(10):e0164367. doi: 10.1371/journal.pone.0164367 (PMC5055312; doi:10.1371/journal.pone.0164367)
Supplement: S1 Table — We expressed Protein A-Chit1-V5-His in E. coli or Chit1-V5-His in CHO cells and measured their chitinolytic activity as described in Materials and Methods. (DOC) [file pone.0164367.s005.doc]

| **Fraction** | **Total activity (mU)** |
| --- | --- |
| Periplasm 1 (Peri 1) | 116.2 |
| CHO-expressed Chit1 | 451.9 |
